# Preclinical Studies of Canagliflozin, a Sodium-Glucose Co-Transporter 2 Inhibitor, and Donepezil Combined Therapy in Alzheimer’s Disease

**DOI:** 10.3390/ph16111620

**Published:** 2023-11-16

**Authors:** Gabriela Dumitrita Stanciu, Daniela Carmen Ababei, Carmen Solcan, Veronica Bild, Andrei Ciobica, Sorin-Ioan Beschea Chiriac, Loredana Maria Ciobanu, Bogdan-Ionel Tamba

**Affiliations:** 1 Advanced Research and Development Center for Experimental Medicine (CEMEX), Grigore T. Popa University of Medicine and Pharmacy, 16 Universitatii Street, 700115 Iasi, Romania; gabriela-dumitrita.s@umfiasi.ro (G.D.S.); bogdan.tamba@umfiasi.ro (B.-I.T.); 2 Pharmacodynamics and Clinical Pharmacy Department, Grigore T. Popa University of Medicine and Pharmacy, 16 Universitatii Street, 700115 Iasi, Romania; 3 Faculty of Veterinary Medicine, “Ion Ionescu de la Brad” University of Life Sciences, 700490 Iasi, Romania; 4 Physiology Department, Grigore T. Popa University of Medicine and Pharmacy, 16 Universitatii Street, 700115 Iasi, Romania; 5 Alexandru Ioan Cuza High School, 37 Ion Creanga Street, 700317 Iasi, Romania; 6 Department of Pharmacology, Clinical Pharmacology and Algesiology, Grigore T. Popa University of Medicine and Pharmacy, 16 Universitatii Street, 700115 Iasi, Romania

**Keywords:** Alzheimer’s disease, SGLT2i, canagliflozin, donepezil, acetylcholinesterase inhibitor, mTOR

## Abstract

The incidence of neurodegenerative diseases, such as Alzheimer’s disease (AD), is continuously growing worldwide, which leads to a heavy economic and societal burden. The lack of a safe and effective causal therapy in cognitive decline is an aggravating factor and requires investigations into the repurposing of commonly used drugs. Sodium-glucose co-transporter 2 inhibitors (SGLT2i) are a new and efficient class of hypoglycemic drugs and, due to their pleiotropic effects, have indications that go beyond diabetes. There is emerging data from murine studies that SGLT2i can cross the blood–brain barrier and may have neuroprotective effects, such as increasing the brain-derived neurotrophic factor (BDNF), reducing the amyloid burden, inhibiting acetylcholinesterase (AChE) and restoring the circadian rhythm in the mammalian target of rapamycin (mTOR) activation. The current study investigates the effect of an SGLT2i and donepezil, under a separate or combined 21-day treatment on AD-relevant behaviors and brain pathology in mice. The SGLT2i canagliflozin was found to significantly improve the novelty preference index and the percentage of time spent in the open arms of the maze in the novel object recognition and elevated plus maze test, respectively. In addition, canagliflozin therapy decreased AChE activity, mTOR and glial fibrillary acidic protein expression. The results also recorded the acetylcholine M1 receptor in canagliflozin-treated mice compared to the scopolamine group. In the hippocampus, the SGLT2i canagliflozin reduced the microgliosis and astrogliosis in males, but not in female mice. These findings emphasize the value of SGLT2i in clinical practice. By inhibiting AChE activity, canagliflozin represents a compound that resembles AD-registered therapies in this respect, supporting the need for further evaluation in dementia clinical trials.

## 1. Introduction

Alzheimer’s disease (AD), defined as a progressive neurodegenerative disorder, is clinically characterized by severe memory loss and the impairment of various cognitive functions. The number and proportion of people with Alzheimer’s and other dementias is expected to continue to grow from 55 to 152 million by the year 2050, because the risk of dementia increases with advancing age [1]. More than a century after its discovery, with amyloid hypothesis as one of the well-known hallmarks of the disease, AD still remains a daunting medical and socio-economical challenge [2,3]. Adherent to the amyloid hypothesis, more and more reports indicated that abnormal accumulation and aggregation of amyloid beta (Aβ) peptides plays a major role in triggering a cascade of pathological events leading to the clinical syndrome of AD [4,5]. Consequently, much of the therapeutics have focused on the extracellular deposits of the Aβ protein and intracellular accumulation of neurofibrillary tangles of the tau protein [2,6,7]. As such, the available drugs, four acetylcholinesterase inhibitors (donepezil, galantamine, rivastigmine and tacrine) and one N-methyl-D-aspartate (NMDA) receptor antagonist (memantine), aim to improve the memory by inhibiting the acetylcholinesterase (AChE) enzyme [8,9,10]. Recently, two anti-amyloid antibodies targeting aggregated (Aducanumab) and protofibril (Lecanemab) forms of Aβ have been approved by the Food and Drug Administration (FDA), but with only small clinical benefits [11,12,13,14]. However, AD is not a consequence of a single factor like AChE, but rather is a multifactorial condition, and this needs to be considered when designing a drug [14]. Other factors, such as chronic disturbances of glucose metabolism, disrupted integrity of the blood–brain barrier, increased inflammation, mitochondrial dysfunction and intracellular oxidative stress, play a significant role in memory and cognitive decline [3,7,15]. The lack of safe and effective agents capable of impacting this devastating disease and its progression is concerning, and invites the repurposing of commonly used drugs and the expanded testing of new mechanistic hypotheses to attack the disease from different angles.

One such potential repurposing involves compounds including sodium-glucose co-transporter 2 inhibitors (SGLT2i) represented by canagliflozin, empagliflozin, dapagliflozin and ertugliflozin, also called flozins or gliflozins [16,17]. SGLT2i are a class of antihyperglycemic agents that modulate sodium-glucose transport proteins, expressed primarily in segments 1 and 2 of the renal proximal convoluted tubules, lowering the renal threshold for glucose, reducing the reabsorption of filtered glucose and promoting urinary glucose excretion [18,19]. These drugs represent a new therapeutic strategy for diabetes, cardiovascular and renal diseases [20]. The mechanistic pathways and molecular targets of these compounds are not yet completely defined, making it even more significant to advance further insight into the action of SGLT2i, especially by searching for new mechanisms that have not yet been considered.

In addition, a wealth of evidence indicates a strong participation of SGLT2i in improving cognitive functions, by inhibiting AChE activity and increasing the acetylcholine levels [21,22,23,24]. Furthermore, these compounds improve peripheral insulin sensitivity, as well as brain signaling, which is impaired in the AD pathogenesis [25]. Recently, a clinical trial confirmed improved insulin sensitivity of the hypothalamus in prediabetes patients after empagliflozin treatment [26]. SGLT2i therapy prevents the formation of advanced glycation end products (AGEs) and its receptor RAGE (receptor for AGE), and blocks the RAGE ligand binding associated with Aβ glycation [27]. In a murine model of AD crossed with a diabetes model of leptin receptor deficiency (db/db), oral SGLT2i empagliflozin therapy was associated with a significant reduction in AD pathology, including the amyloid plaque density and soluble Aβ levels. In addition, these findings were correlated with a lower level of brain atrophy, neuronal loss, cortex microhemorrhages and brain inflammation driven by microglia [28]. According to Lin et al. [29], db/db mice treated with empagliflozin had a suggestively higher level of brain-derived neurotrophic factor (BDNF), which was associated with better cognitive functions. BDNF is a well-studied member of the neurotrophin family, with a crucial role in facilitating nerve growth and maturation, modulating neurotransmission and plasticity. In the context of AD, BDNF depletion is linked to Aβ deposition, neuroinflammation, tau phosphorylation and neuronal apoptosis [30]. The increase in BDNF may explain the results by Sa-Nguanmoo et al. [31] from obese rats treated with dapagliflozin, which showed an improvement in hippocampal synaptic plasticity. Canagliflozin also decreased obesity-associated neuroinflammation in the hypothalamus [32], improved the insulin response and was partially associated with reduced phosphorylation of S6 kinase in microglia in aged mice [33]. Canagliflozin is even called the “dual inhibitor of SGLT2i and AChE”, as its molecular structure enables acetylcholinesterase inhibition [22]. Moreover, SGLT2i strongly promote anti-inflammatory M2 macrophage polarization [34]. In patients taking canagliflozin, the serum level of interleukin-6 (IL-6) decreased by approximately 27% after 2 years of treatment [35]. Pleiotropic anti-inflammatory (the reduction of tumor necrosis factor alpha (TNF-α), IL-6, interleukin 1β (IL-1β), monocyte chemoattractant protein-1 (MCP-1), intercellular adhesion molecule 1 (ICAM-1), also known as CD54) and anti-oxidative properties of SGLT2i were correlated with the beneficial effects on cellular metabolism, including the activation of sirtuin 1 (SIRT1) [36]. There is a concept on the “state of fasting mimicry”, which assumes that therapy with SGLT2i improves the general condition of cells by determining the transcriptional changes that occur during starvation, and include SIRT/activated protein kinase (AMPK) activation, along with mTOR suppression [30,37]. Chronic unrestrained mTOR activation may be behind AD metabolic dysfunction, causing the breakdown of the blood–brain barrier (BBB) via endothelial cell dysfunction, as well as leading to tau hyperphosphorylation, amyloid plaques formation and aggregation in the brain [38]. Thus, the question of whether SGLT2i canagliflozin has protective effects in neurodegeneration, in diabetes-free mice, remains open. In this study, we evaluated the possible neuroprotective effect of canagliflozin under separate or combined 21-day treatment, with a specific focus on AD-relevant behaviors and cognitive function.

## 2. Results

### 2.1. Nootropic and Anti-Amnesic Effects of Canagliflozin and Donepezil, under Separate or Combined Therapy

**Figure 1:**
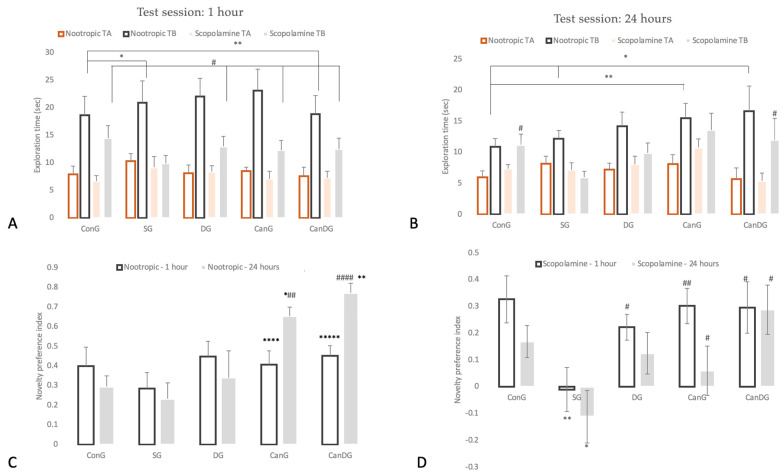
Behavioral analysis for novel object recognition test (NORT). Panel: TA, denotes familiar object; TB, denotes the novel object. (**A**) Data are represented as exploration time (seconds) in the NORT, test session for the nootropic model compared to the scopolamine model after an interval of 60 min following the training period; (**B**) represents the graph plot for the exploration time (seconds) in the NORT, test session for the tested models (nootropic and scopolamine) after an interval of 24 h following the training period; (**C**) represents the graph plot for the novelty preference index for the nootropic model; (**D**) the novelty preference index for the scopolamine model. Data are expressed as mean ± SEM, and statistical analysis using a one-way ANOVA, ∗ *p* < 0.05 vs. ConG, ∗∗ *p* < 0.01 vs. ConG and ∗∗∗∗ *p* < 0.0001 vs. ConG; ∗∗∗∗∗ *p* < 0.00001 vs. ConG; # *p* < 0.05 vs. SG, ## *p* < 0.01 vs. SG and #### *p* < 0.0001 vs. SG. ConG: control group; SG: negative control group—scopolamine; DG: positive control group—donepezil; CanG: canagliflozin group; CanDG: canagliflozin–donepezil group.

To evaluate the neuroprotective and memory-enhancing effects of SGLT2i canagliflozin, donepezil and a combination of both, on a scopolamine-induced learning and memory deficits animal model, we performed the novel object recognition test (NORT), which assesses short- and long-term recognition memory. The findings obtained from NORT on the drug therapy nootropic activity are illustrated in Figure 1A–C and were measured following 12 days of pretreatment. On the training day, the % of time spent exploring each of the familiar objects did not differ between the control and the treated animals (*p* > 0.05). On the test day, DG, CanG and CanDG mice explored the novel object significantly more than the familiar one (*p* < 0.01). The groups pretreated with canagliflozin and canagliflozin + donepezil showed an increased novelty preference index after an interval of 60 min (CanG *p* < 0.0001 vs. ConG, CanDG *p* < 0.00001 vs. ConG) or after 24 h following the training period (CanG *p* < 0.01 vs. SG and CanDG *p* < 0.0001 vs. SG). In the chronic scopolamine model, the NORT showed a reduction in the novelty preference index percentage for the negative group (SG, 3 mg/kg), as shown in Figure 1D. Moreover, the novelty preference index percentage for CanG and CanDG was comparable with the donepezil group (0.65 mg/kg).

We found no evidence to suggest that the chronic administration of canagliflozin and donepezil had a significant impact on anxiety-like behavior. In the elevated plus maze (EPM) test, by evaluating the percentage of time spent in the open arms of the maze, there were no significant differences between the treated animals and the controls (*p* > 0.05, one-way ANOVA) in the nootropic phase compared to the anti-amnesic phase.

### 2.2. Paraclinical Evaluation: Biochemistry Assay and Immunohistochemistry Analysis

#### 2.2.1. Biochemistry Profile

The serum biochemical parameters resulting from separate or combined therapy with SGLT2i canagliflozin and donepezil, graded as the mean of the group ± SEM, are presented in Table 1. The standardized biochemical panel was, thus, chosen for the indirect quantification of the liver and kidney function, the main organs responsible for the metabolism of these compounds. The data presented in Table 2 shows that creatinine, aspartate aminotransferase, alanine aminotransferase, total cholesterol and albumin changed under drug therapy, without statistical differences. Nevertheless, SGLT2i canagliflozin, donepezil and the combination of the two drugs significantly improved the levels of glucose (CanG—*p* < 0.05, CanDG—*p* < 0.01) compared with ConG, and urea (CanG—*p* < 0.01, CanDG—*p* < 0.001) compared with the scopolamine group.

In order to clarify the potential mechanisms that could impact cognitive impairment in scopolamine mice, the effect of drug therapy on AChE activity was investigated. The scopolamine treatment increased the AChE activity compared to that of the control group, whereas the drug groups (CanG and DG groups) significantly decreased the AChE activity. Moreover, the combination group (CanDG mice) displayed a stronger reversal effect than the other two administration groups and had a significant difference compared to the SG group (*p* < 0.05).

#### 2.2.2. Immunohistochemistry Analysis

To assess the implications of the drug therapy on mice with scopolamine-induced amnesia, we analyzed the expression of some parameters using immunohistochemistry at the brain level, such as the muscarinic acetylcholine receptor M1 (M1mAChR), the vascular endothelial growth factor A (VEGF-A), cyclooxygenase-2 (COX2), the mammalian target of rapamycin (mTOR), the glial fibrillary acidic protein (GFAP), the major histocompatibility complex class II (MHC II), the cluster of differentiation 68 (CD68), the nuclear factor erythroid 2-related factor 2 (Nrf2) and p65. The quantitative immunohistochemistry (IHC) analysis of the biomarkers is shown in Table 2.

**Figure 2:**
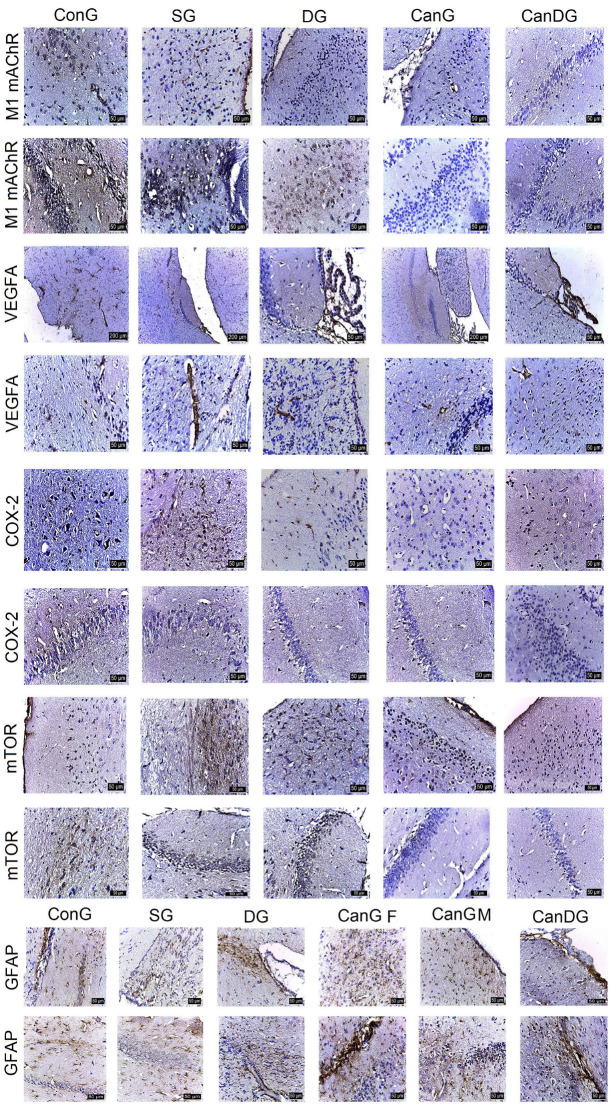
Representative images of the effect of treatments on the expression of muscarinic acetylcholine receptor M1 (M1mAChR), vascular endothelial growth factor A (VEGF-A), cyclooxygenase-2 (COX2) and the mammalian target of rapamycin (mTOR) in the studied groups. The M1mAChR marker showed positivity in the subcortical area and the hippocampus in the ConG. M1mAChR expression was lower to moderate in mice in the CanG and CanDG groups compared to the scopolamine group. VEGF-A labelled the endothelial cells in the capillaries in all areas of the nervous system, including the ciliary processes. The expression of the COX2 and mTOR markers registered close reactivity, more intense in the scopolamine mice and less in the CanG, DG and CanDG mice. The positive expression was recorded in the cortical, subcortical and hippocampal areas for the glial fibrillary acidic protein (GFAP). The denser cell population was observed in the control, scopolamine, donepezil groups and in the females exposed to canagliflozin. In the males treated with canagliflozin and in the CanDG mice, a reduction in the GFAP positive cell population was observed. ConG: control group; SG: negative control group—scopolamine; DG: positive control group—donepezil; CanG: canagliflozin group; CanDG: canagliflozin–donepezil group; SGLT2i: sodium-glucose cotransporter 2 inhibitor; M1mAChR: muscarinic acetylcholine receptor M1; VEGF-A: vascular endothelial growth factor A; COX2: cyclooxygenase-2; mTOR: mammalian target of rapamycin; GFAP: glial fibrillary acidic protein.

M1mAChR showed low to moderate expression in the hippocampus in mice treated with canagliflozin, or canagliflozin plus donepezil, in comparison with the scopolamine group (Figure 2). VEGF-A positively marked the endothelial cells in the capillaries in the gray and white matter and in the choroid plexuses in all the groups in the study (Figure 2). COX2 expression (Figure 2) was positive in the scopolamine group. In the other groups (DG, Can and CanDG), COX2 expression was reduced and even absent in the examined areas. The positively labeled mammalian target of rapamycin (mTOR) showed a positive expression in the ConG, SG and DG groups in the subcortical, periventricular and hippocampal areas. In the other groups (CanG and CanDG), the expression was reduced in the cortical area and was frequently absent in the hippocampus (Figure 2). The GFAP marker of glial cells was observed in a higher proportion in the control, scopolamine, donepezil groups and in females exposed to canagliflozin. The positive glial cells (astrocytes) were larger than microglia, presenting thicker and shorter extensions in the subcortical, periventricular area and in the immediate vicinity of the hippocampus. In males treated with canagliflozin and CanDG mice, a reduction in the positively marked glial population was found (Figure 2).

**Figure 3:**
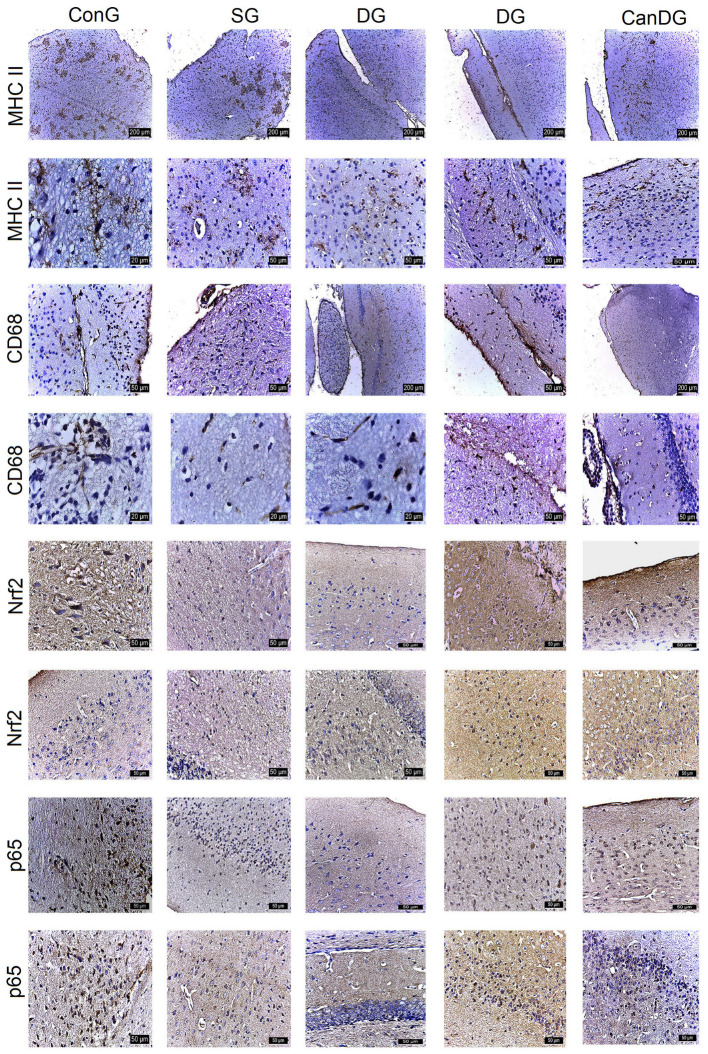
The different expressions of the major histocompatibility complex class II (MHC II), cluster of differentiation 68 (CD68), nuclear factor erythroid 2-related factor 2 (Nrf2) and p65 expression in the control and experimental groups. MCH II labeled microglia were small with numerous short, branched extensions, the cells being grouped in the periventricular zone. CD68 positive microglia had an amoeboid appearance, present especially in the periventricular area. The frequency of the cell populations was lower in the CanG and CanDG groups. An inverse expression of the Nrf2 and p65 markers was observed in the areas studied. MHC II: major histocompatibility complex class II; CD68: cluster of differentiation 68; Nrf2: nuclear factor erythroid 2-related factor 2; ConG: control group; SG: negative control group—scopolamine; DG: positive control group—donepezil; CanG: canagliflozin group; CanDG: canagliflozin–donepezil group.

MHC II registered overexpression in the periventricular zone in the scopolamine group. An average expression was observed in the control mice and in the donepezil-treated animals. Positively labeled microglia showed numerous short, thin, branched extensions with a small ellipsoidal body, and could be seen clustered. MHC II expression was reduced in the CanG and CanDG mice (Figure 3). CD68 showed expression in the control and scopolamine groups, and less in the animals treated with canagliflozin and donepezil under separate or combined therapy. Microglia, with the role of macrophages, had an amoeboid appearance, without extensions, being present in the periventricular area and hippocampus (Figure 3).

Nrf2 showed positivity in some neurons in the subcortical area and negative expression in the hippocampus in the SG group. In the mice exposed to donepezil, moderate positivity was found in some cells in the cerebral cortex, subcortical and hippocampus area. The groups treated with canagliflozin or canagliflozin + donepezil expressed positivity both in the cortical area and in the hippocampus (Figure 3). P65 showed moderate positive expression in the hippocampus in the group exposed to scopolamine and was reduced to a negative expression in the other groups. In the CanG and CanDG mice, rare positive cells appeared in the cerebral cortex, the sub-regions of the cerebral cortex and the hippocampus (Figure 3).

## 3. Discussion

The current study aims to verify whether canagliflozin as compared to donepezil, as a reference drug, is able to influence Alzheimer’s disease-like conditions by modulating the cholinergic pathway and inhibiting SGLT2i. A mouse model of learning and memory deficits treated with intraperitoneal scopolamine was adopted to verify this hypothesis. In neuroscience-related research, scopolamine is often used to induce cognitive disorders in experimental models as it readily permeates the blood–brain barrier. In the context of Alzheimer’s disease, its effects include causing cholinergic dysfunction and increasing amyloid-β deposition, both of which are hallmarks of the disease [39].

In our experiment, the NORT and EPM were applied as behavioral models to assess the cognitive status of the mice. In AD research, the NORT is particularly relevant because it allows the evaluation of visual recognition memory, an early marker in the disease progression and diagnosis [40]. On the other hand, the EPM is a behavioral test used to study long-term spatial memory [41]. The behavior measurements demonstrated nootropic activity in both the novelty preference index and percentage of time spent in the open arms of the maze under SGLT2i canagliflozin therapy, in the NORT and EPM tests, respectively. Based on these results, it can be suggested that canagliflozin represents a nootropic drug that may act as a natural cognitive enhancer. Nootropic drugs, also known as “smart compounds”, can be used as a supportive therapy in patients with Alzheimer’s disease, schizophrenia, stroke, vascular or senile dementia [42].

Scopolamine, a nonselective muscarinic cholinergic receptor antagonist associated with cholinergic dysfunction leading to performance deficits in memory and learning, has been widely used to evaluate potential therapeutic compounds for AD therapy [43]. Consequently, in this study, scopolamine was administered to mice for 9 days to induce cholinergic neurodegeneration, accompanied by cognitive deficits [44]. Following scopolamine administration, the scopolamine-treated mice showed a reduction in the novelty preference index percentage. Pretreatment with canagliflozin or canagliflozin plus donepezil ameliorated scopolamine-induced memory impairment, with the recognition index being greater in comparison with the scopolamine-treated group and comparable to that of the donepezil mice. These findings highlighted that canagliflozin was as effective as the donepezil-treated group. Moreover, the results showed that canagliflozin therapy attenuated amnesic behavior in the EPM, but it was insignificant. Therefore, these outcomes suggest that canagliflozin had an anti-amnesic effect in the scopolamine model, partly via enhancing cholinergic neurotransmission.

In AD patients, dysfunction of the cholinergic system is evidenced by increased activity of AChE, an important enzyme that hydrolyses acetylcholine (ACh), an essential neurotransmitter implicated in memory and learning; so, we therefore investigated the AChE-inhibitory effect of canagliflozin. An increase in AChE activities is reported in the present research in the negative control group (SG mice), as a biomarker of scopolamine- induced cognitive impairment. Similar results were reported by Weon et al. [45] and Bhuvanendran et al. [44]. In the AChE activity assay, mice treated with canagliflozin or donepezil decreased the AChE activity and the co-therapy with canagliflozin and donepezil (CanDG mice) displayed a stronger effect. These results indicated that canagliflozin under separate or combined therapy ameliorated the scopolamine-induced memory deficit by increasing the cholinergic activity through the inhibition of the AChE activity. These data are consistent with the decrease in the M1mAChR expression in the cortex and hippocampus of mice in the CanG and CanDG groups, compared to the ConG. In line with this notion, the above-mentioned feature of the memory impairment model has also been stated in other previous studies [46,47]. Recent research involving obese diabetic rats demonstrated inhibition of AChE under canagliflozin gavage therapy [47], which is in agreement with our result. In an enzoinformatics study, canagliflozin was strongly suggested as a dual inhibitor of SGLT2i and AChE [22], even though there is no similarity between the transport channel of SGLT2i and the catalytic site of AChE. Our finding about CanG and CanDG mice, which exhibited a significant increase in hippocampus M1mAChR as compared to the SG mice, may support this suggestion.

The results from the biochemical analysis showed normal values for creatinine, aspartate aminotransferase, alanine aminotransferase, total cholesterol and albumin values considering the age of the animals (40 weeks at the beginning of the study) [48], in all groups. It is well-known that the C57BL/6 mouse strain is generally suggested to be the best strain for studying metabolic disease, because they are more prone to developing diabetes and diet-induced obesity. They are also the base strain for the ob/ob mouse (hyperphagic, obese, hyperinsulinemic and hyperglycemic). Consequently, there are probably many factors (nutrition, vascular volume, hormonal changes, etc.) besides colloid osmotic pressure that contribute to the changing levels of the serum proteins throughout the life span of the mice [49,50]. Canagliflozin monotherapy or in combination with donepezil significantly improved the blood glucose, urea and total protein levels compared with the control animals. Canagliflozin targets the sodium-glucose co-transporter 2, the major glucose transporter in the kidney, responsible for the reabsorption of 90% of the glucose from primary urine. Inhibition of SGLT2 decreases glucose reabsorption and, thus, increases urinary glucose excretion, leading to a reduction in both fasting and postprandial hyperglycemia, preventing glucotoxicity and hyperglycemia-induced damage [51,52].

Data from the behavioral test served as a positive confirmation of the results obtained from the immunohistochemical evaluation. As previously mentioned, increased AChE activity in AD exacerbates Aβ plaque formation, which in turn activates astrocytes and upregulates GFAP, an indicator of neuroinflammation [53]. Canagliflozin administration counteracted the scopolamine-induced elevation of GFAP expression in males treated with canagliflozin or canagliflozin plus donepezil, whereas in females exposed to canagliflozin we observed only a decrease in the hypothalamic neuroinflammation. Moreover, MHC II and CD68 showed expression in the scopolamine group and were less observed in the animals treated with canagliflozin and donepezil under separate or combined therapy. Microglia, with the role of macrophages, were characterized by an amoeboid appearance, without extensions, being present in the periventricular area and hippocampus. Age-related increases in cerebral pro-inflammatory cytokines are considered to be detrimental in both humans and mice, correlating with deficits in cognitive function [54]. In support of our findings, a recent study has reported that aged male UM-HET3 mice generated more robust neuroimmune responses than aged females. Thus, canagliflozin therapy showed substantial reductions in age-associated hypothalamic gliosis, with a decrease in inflammatory cytokine production by microglia [33]. Moreover, our findings are in agreement with a prior study [55] and imply that scopolamine is upregulated in an inflammatory cascade via astrocytic activation.

In the current study, canagliflozin treatment was associated with the presence of VEGF-A expression in the gray and white matter endothelial cells and choroid plexuses in all groups. In the context of aging and AD, despite the complexity and mixed evidence reported for both up- and downregulation of the VEGF-A gene and protein expression in the brain, fluid cerebrospinal and blood [56], there is increasing evidence that the VEGF-A gene plays a critical role in reducing glucose uptake [57], with neuroprotective effects or even represents, according to some authors, a potential biomarker of neuroinflammation [58].

As part of the cholinergic lesion, COX2 activity is known to be increased in the brain of AD patients and symptoms severity correlates positively with both COX2 activity and increased Aβ expression [59]. Consistent with previous findings, our data demonstrated that COX2 expression was increased in the hippocampus of the scopolamine-treated mice. In the other groups (DG, Can and CanDG), COX2 expression was reduced and even absent in the examined areas.

Canagliflozin reduced the expression of mTOR in the cortical area and was frequently absent in the hippocampus of aged mice in the CanG and CanDG groups compared with the SG animals. In support of our findings, several studies have reported altered mTOR activity in AD brain and AD mouse models, supporting the notion that aberrant mTOR activity may be one of the main events contributing to the onset and progression of AD hallmarks [60,61]. This aberrant activation of mTOR in mice correlates with dysfunction of energy metabolism, extensive amyloid plaque deposits, tau protein hyperphosphorylation and increased BBB permeability [60,62,63]. Taken together, these data are consistent with the hypothesis that SGLT2i downregulates mTOR expression, mitigating perturbed cellular metabolic profiles.

From a neuropathological point of view, a key feature in AD is the accumulation of reactive oxygen species (ROS), which leads to an overall increase in oxidative damage. The Nrf2 is a major regulator of the antioxidant response in cells and neuroinflammation [64]. Nrf2 activation increases the autophagy function. However, in AD pathology, the accumulation of Aβ and tau causes a decrease in the Nrf2 levels, diminishing the antioxidant response. Consequently, lower Nrf2 levels contribute to the further deposition of Aβ and tau by impairing their autophagy-mediated turnover [65]. Consistent with these findings, our data demonstrated that Nrf2 showed positivity in some neurons in the subcortical area and negative expression in the hippocampus in the SG mice, while the groups treated with canagliflozin or canagliflozin + donepezil expressed positivity both in the cortical area and in the hippocampus.

Pretreatment with canagliflozin reduced p65 expression in the cerebral cortex, the sub-regions of the cerebral cortex and the hippocampus in the CanG and CanDG mice compared with the scopolamine group; this suggests that canagliflozin ameliorated the cognitive deficit via suppression of the inflammatory cascade, probably through its antioxidant and anti-inflammatory effects. This finding is consistent with other studies [66,67].

The accurate extrapolation of animal data directly to humans may not be fully guaranteed due to interspecies variation in anatomy and physiology, but they can aid researchers to investigate other mechanisms that may underlie the neuroprotective effect of canagliflozin. In addition, appropriate target engagement and safety studies should help define clinically meaningful doses and therapeutic windows. Clinical studies to investigate the compound’s validity to prevent or slow down the progression of AD are additional research directions that can be pursued, following the current investigation.

## 4. Materials and Methods

### 4.1. Animal Care

Both female and male C57BL mice (n = 50, 40 weeks old; Cantacuzino Institute, Bucharest, Romania) were used. The animals were housed in the animal facility at the Advanced Research and Development Center for Experimental Medicine “Prof. Ostin C. Mungiu”-CEMEX, in individually ventilated cages (IVCs) and maintained in standard husbandry conditions: controlled room temperature (20 ± 4 °C), relative humidity (50 ± 5%) and stress light–dark cycle; with ad libitum access to water and standard laboratory chow.

The experimental protocol and procedures followed the European Community Guidelines (Directive 2010/63/EU) and Romanian law (Low no. 43/2014) on the protection of animals used for scientific purposes, and were reviewed and approved by the Ethical Committee at ‘‘Grigore T. Popa’’ University of Medicine and Pharmacy of Iasi (no. 71/22.04.2021) and the National Sanitary Veterinary and Food Authority (no. 36/26.05.2021).

### 4.2. Drugs

Scopolamine hydrobromide (Sigma-Aldrich, St. Louis, MO, USA) (assay > 90%) was dissolved in 0.9% saline. Donepezil was supplied as “Aricept” 10 mg orodispersible tablets, purchased from Pfizer, and canagliflozin was supplied as ”Invokana” 100 mg tablets, manufactured by Janssen Pharmaceuticals. Donepezil and canagliflozin were grinded to a powder and an appropriate amount was suspended in 0.5% carboxymethyl cellulose sodium (CMC-Na) salt solution. Compound doses were selected using other Alzheimer’s disease modeling methods or the body surface area conversion factor. Thus, a dose of 0.65 mg/kg was used for donepezil (clinical equivalent) [68], 3 mg/kg for scopolamine [45] and 10 mg/kg for canagliflozin [69].

### 4.3. Experimental Design

**Figure 4:**
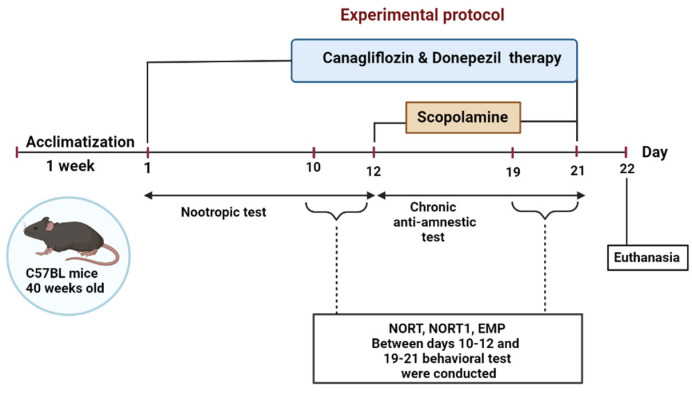
Schematic representation of the overall experimental procedure. For 21 consecutive days mice were treated with either donepezil, canagliflozin, donepezil+ canagliflozin or the vehicle solution using gavage and with intraperitoneal injections with scopolamine for the last 9 days of the study. Behavioral tests were performed between days 10-12 and 19-21; as indicated in the inset. NORT: novel object recognition test; EPM: elevated plus maze.

After 7 days of laboratory acclimatization (when the mice were habituated to the presence of the researchers and handled before testing), the animals were divided into five groups (n = 10/group) and treated for three weeks. (1) The control group (ConG) was treated daily using gavage 0.5 mL/100 g CMC-Na 0.5%. (2) The negative control group—scopolamine (SG), was treated using intraperitoneal injections of scopolamine (3 mg/kg) for 9 days (day 13 to day 21). (3) The positive control group—donepezil (DG) was treated daily using gavage (0.65 mg/kg) and injected with scopolamine in the last 9 days of the study. (4) The canagliflozin group (CanG) was treated daily using gavage canagliflozin (10 mg/kg) and injected with scopolamine in the last 9 days of the study. (5) The canagliflozin–donepezil group (CanDG) was treated daily with canagliflozin plus donepezil (10 mg/kg + 0.65 mg) and injected with scopolamine in the last 9 days of the study. The timeframe of the experiments is shown in Figure 4.

Cognitive performance evaluation was performed during the light phase, using the novel object recognition test (NORT) and the elevated plus maze (EPM), and was divided into two stages to test the nootropic and anti-amnesic activities of the canagliflozin. To assess the nootropic activity, all the animals were pretreated using gavage for 12 days. The mice were then subjected to a battery of behavioral tests from day 10 to day 12 for the NORT and EPM. Cholinergic neurodegeneration, along with cognitive deficits, was induced in all the groups except the control one, with daily intraperitoneal injections of scopolamine during the last 9 days of the study. Behavioral tests were carried out on days 19–21, half an hour after scopolamine administration.

For the NORT task, an open field arena (50 × 50 × 50) composed of black acrylic material was used. The test involved three sessions: (a) habituation, (b) training and (c) test. On the first day, the mice were permitted to familiarize themselves with the arena without the presence of an object or any stimulus for about 5 min, under the same environmental and lighting conditions. In the training period, each mouse was placed in the arena for 5 min and allowed to freely explore two identical objects (familiar objects, cultured flask filled with water) and the environment. The test session comprised the assessment of short-term and long-term memory, after an interval of 60 min and 24 h following the training period, respectively [70]. The mice were placed inside the open field arena with a novel object (a Lego toy similar in height to the flask) and a familiar object, and left to explore the objects for 5 min. Between every run the objects and arena were cleaned with 70% ethanol to minimize any olfactory clues. The exploratory behavior was recorded and evaluated using the video tracking software Smart 3.0 Basic Pack/Smart 3.0 SUPER (Harvard Apparatus). The activity was quantified as the time (seconds) the mouse spent investigating each object (direct approaches ≤ 1 cm distance were considered). The number of explorations, which included sniffing the object or touching the object with its nose and/or forepaws [71], was counted. Exploration of each object was quantified as the novelty preference index (NPI), calculated as (TB − TA)/(TB + TA), where TA corresponds to the time spent exploring the familiar object and TB is the time spent exploring the novel object, during the test phase of the NORT [71].

The anxiety responses of mice were assessed using the EPM. The animals were placed in the intersection of the four arms (two open and two enclosed) of the elevated plus maze, shaped like a plus sign and elevated 50 cm above the floor, immediately after being tested in the NORT during the test day, and their behavior was recorded for 5 min [72]. Anxiety-like behavior was measured using the total time spent in the open arms [73].

### 4.4. Paraclinical Evaluation: Biochemistry Assay and Immunohistochemistry Analysis

All the mice were euthanized (neck dislocation under anesthesia) after the completion of the behavioral tests, and a cardiac puncture was performed to sample 1 mL of terminal blood in 3 mL clot activator vacutainer tubes for biochemistry profiling and the acetylcholinesterase activity assay. In each group, six mouse brains (three male and three females) were collected. Each brain was sampled and fixed in 10% formalin for a detailed immunohistochemical stain analysis.

Biochemistry analysis was used to investigate the implications of SGLT2i canagliflozin and donepezil, under separate or combined 21-day treatment, on the primary organs responsible for drug metabolism (e.g., kidney and liver). A series of biochemical parameters (creatinine, aspartate transaminase (AST), alanine transaminase (ALT), total cholesterol, glucose, albumin, urea and total protein) were used, as previously described by our team [74].

Moreover, 30 min after harvesting, the vacutainer tubes were centrifuged at 1500× *g* for 15 min at 4 °C; the separated serum samples were then subjected to biochemistry analysis using an ACCENT-200 analyzer (PZ Cormay, Warsaw, Poland). The acetylcholinesterase activity was conducted, as mentioned by Al-Hazmi et al. [46], and measured by commercially available kits (Colorimetric, ab138871). All the experimental steps were carried out according to the manufacturer’s protocol, using a microplate reader to measure the absorbance at their respective absorption wavelengths.

The immunohistochemical (IHC) staining was performed according to previously described protocols [75,76] with modifications, using the antibodies listed in Table 3. The brain from each mouse was processed using the ExcelsiorTM AS Tissue Processor (Epredia Holdings Ltd., Portsmouth, NH, USA) and embedded in a single paraffin wax block. All the embedded paraffin blocks were then sectioned using a semi-automatic microtome CUT 5062 (SLEE medical GmbH, Nieder-Olm, Germany), at a 4 μm cutting thickness. Three sections for each animal were then transferred onto a microscope slide and stained using hematoxylin and eosin (H&E) standard staining protocol. Subsequently, all the H&E-stained tissue microscope slides were examined using light microscopy using an Aperio AT2 DX slide scanner (Leica 557 GmBh, Berlin, Germany), at a 400× magnification scale. Photomicrographs were then analyzed and compared to the control by a veterinary histopathologist.

Tissues were used for the detection of muscarinic acetylcholine receptor (M1 mAChR) expression, vascular endothelial growth factor A (VEGFA), cyclooxygenase-2 (COX2), the mammalian target of rapamycin (mTOR), the glial fibrillary acidic protein (GFAP), major histocompatibility complex class II (MHCII), the cluster of differentiation 68 (CD68), nuclear factor erythroid 2-related factor 2 (Nrf2) and p65.

### 4.5. Data Analysis and Statistics

All data sets were expressed as mean ± standard error of the mean (SEM) and analyzed in Prism 7.0 (GraphPad Software 8, Boston, MA, USA). The novelty preference index for the NORT test and the percentage of time spent in the open arms of the maze, as a measure of anxiety-like behavior in the EPM test, were statistically analyzed using a one-way analysis of variance (ANOVA). The *p*-values of ∗ *p* < 0.05, ∗∗ *p* < 0.01, ∗∗∗ *p* < 0.001 and ∗∗∗∗ *p* < 0.0001 were considered as statistically significant. All the experimental groups were compared with the ConG and SG groups.

## 5. Concluding Remarks

Our analysis highlights that the memory-enhancing effect of canagliflozin may result from the anticholinesterase activity in the brain areas, SGLT2 inhibition, anti-inflammatory properties, the reduction in oxidative stress, and the restoration of a balance between the catabolism and anabolism. Thus, canagliflozin should be further studied for dual drug therapy. Nonetheless, the results obtained in the present study may reduce the time and cost for the development of drugs associated metabolic disturbances against Alzheimer’s disease.

## Figures and Tables

**Table 1:** Serum biochemistry profile of mice treated with SGLT2i canagliflozin and donepezil, under separate or combined therapy. Values are the mean with their standard errors.

Dose/Parameter	ConG	SG	DG	CanG	CanDG
CRE (mg/dL)	0.29 ± 0.04	0.23 ± 0.03	0.21 ± 0.03	0.17 ± 0.04	0.19 ± 0.05
AST (U/L)	64.76 ± 25.33	78.81 ± 44.82	69.38 ± 52.38	71.03 ± 0.3	61.85 ± 85
ALT (U/L)	53.23 ± 166.98	56.45 ± 106.47	51.01 ± 134.61	52.35 ± 152.80	48.58 ± 89.14
TC (mg/dL)	229 ± 50.96	151.22 ± 15.07	155.5 ± 16.79	125 ± 13.04	121.40 ± 10.30
GLU (mg/dL)	245.3 ± 21.46	183.22 ± 13.42	209.44 ± 12.50	176 ± 17.88 *	170.70 ± 15.71 **
ALB (g/L)	42.71 ± 5.49	45.56 ± 2.36	39.59 ± 4.69	34.62 ± 5.19	32.37 ± 5.49
TP (g/L)	84.87 ± 9.51	74.76 ± 2.58	76.95 ± 2.13	64.98 ± 4.51 ^@^	69.61 ± 2.66 ^@^
UREA (mg/dL)	64.09 ± 11.53	65.78 ± 3.55	53.78 ± 7.06 *^#^*	49.13 ± 7.40 *^##^*	42.43 ± 4.48 *^###^*

CRE: creatinine; AST: aspartate aminotransferase; ALT: alanine aminotransferase; TC: total cholesterol; GLU: glucose; ALB: albumin; TP: total protein; UREA: urea; ConG: control group; SG: negative control group—scopolamine; DG: positive control group—donepezil; CanG: canagliflozin group; CanDG: canagliflozin–donepezil group; * *p* < 0.05 vs. ConG; ** *p* < 0.01 vs. ConG; ^@^ *p* < 0.05 vs. DG; ^#^ *p* < 0.05 vs. SG; ^##^ *p* < 0.01 vs. SG; ^###^ *p* < 0.001 vs. SG.

**Table 2:** Quantitative immunohistochemistry (IHC) analysis of the biomarkers.

Biomarkers	Experimental Animal Groups				
	*ConG*	*SG*	*DG*	*CanG*	*CanDG*
M1 AChR H	+++	++++	+	++	++
M1 AChR SC	++	++++	++	+	+
VEGF-A H	++	++	++	++	++
VEGF-A SC	++	++	++	++	++
COX-2 H	+++	++++	+	+	+
COX-2 SC	++	+++	+	+	+
mTOR H	+++	++++	++	+	+
mTOR SC	++	++++	+++	+	+
GFAP H (females)	+++	+++	+++	+++	+
GFAP H (males)	+++	+++	+++	-	-
GFAP SC (females)	++++	+++	++	+++	+
GFAP SC (males)	+++	++++	+++	-	++
MHC II H	+++	+++	++	+	+
MHC II SC	++	++	+	+	+
CD68 H	+++	+++	++	+	++
CD68 SC	+++	+++	+	+	+
Nrf2 H	+++	++	+	++	++
Nrf2 SC	++	-	++	++++	+++
P65 H	+++	++	+	+	+
P65 SC	+++	+++	+	+	+

Average IHC positive cells/10 fields of 10,000 µm^2^; 1–6 IHC + positive cells; 6–12 IHC ++ positive cells; 12–18 IHC +++ positive cells; 18–24 IHC positive cells ++++; SC: subcortical area; H: hippocampus area.

**Table 3:** Primary and secondary antibodies, with the related dilution used in immunohistochemical analysis.

	Primary Antibody	Dilution	Secondary Antibody	Dilution
1.	mTOR (ab109268)	1:70	Goat anti Rabbit	1:100
2.	P65 (AA 143-158)	1:100	Goat anti Rabbit	1:100
3.	Anti VEGFA (ABIN2788641)	1:250	Goat anti Rabbit	1:1000
4.	GFAP (Cat.nr.173002)	1:500	Goat anti Rabbit	1:500
5.	MHC II (Dako M0746)	1:100	Goat anti Rabbit	1:100
6.	Nrf-2 (WJ3412022B)	1:100	Goat anti Rabbit	1:100
7.	COX2 (ab16701 SP-21)	1:100	Goat anti Rabbit	1:100
8.	M1mAChR (SC365966)	1:250	Goat anti Mouse	1:250

## Data Availability

The datasets used and/or analyzed during the current study are available from the corresponding author upon reasonable request.
